# Influence of Heat Treatment on the Corrosion of the Al–Mg Intermetallic Alloy in Synthetic Seawater

**DOI:** 10.3390/ma19183856

**Published:** 2026-09-10

**Authors:** José Damián Calan-Canche, Alfredo Reda-Cruz, Salatiel Pérez-Montejo, Cristóbal Patiño-Carachure, Sergio Martinez-Vargas, José Enrique Flores-Chan

**Affiliations:** 1 Facultad de Ingeniería, Universidad Autónoma Del Carmen, Campus III, Ciudad del Carmen C.P. 24115, Campeche, Mexico; jodcalan@gmail.com (J.D.C.-C.); cpatino358@gmail.com (C.P.-C.); 2 Mantenimiento Industrial, Universidad Tecnológica de Campeche, San Antonio Cárdenas, Ciudad del Carmen C.P. 24100, Campeche, Mexico

**Keywords:** corrosion, heat treatment, intermetallic, precipitation, aluminum–magnesium alloys

## Abstract

**Highlights:**

- Mg migration promoted by heat treatment in Al−20 wt.% Mg alloy favors the formation of the phases α−Al(Mg) and β−Al_3_Mg_2_
- Heat treatment modulates the β−Al_3_Mg_2_ intermetallic network in Al−20 wt.% Mg alloy
- β−Al_3_Mg_2_ distribution in Al−20 wt.% Mg alloy promotes susceptibility to corrosion
- Anodic (α) and cathodic (β) microgalvanic coupling promotes corrosion

**Abstract:**

In this study, an Al–20 wt.% Mg alloy was synthesized to systematically correlate its electrochemical behavior in synthetic seawater with the microstructural evolution induced by heat treatment at 190, 300, and 350 °C for 6 h. The samples characterized by SEM and XRD reveal a progressive morphological evolution of the β–Al_3_Mg_2_ intermetallic phase. The open circuit potential, the potentiodynamic polarization and the electrochemical impedance spectroscopy showed that this microstructural evolution increased the microgalvanic corrosion, promoting passive-film breakdown, and reducing charge-transfer resistance. The Al–20 wt.% Mg alloy treated at 350 °C exhibited the highest corrosion current density, associated with β–Al_3_Mg_2_. These results demonstrated that controlling beta-phase precipitation through heat treatment provides an effective approach to tune the electrochemical behavior of high-magnesium aluminum alloys.

## 1. Introduction

Aluminum (Al) is the most abundant metallic element and the third most abundant chemical element on Earth’s crust. It occurs naturally in the form of minerals, mainly bauxite due to its high chemical reactivity [1]. Its main properties include low density, high corrosion resistance, and excellent conductivity [2]. Its extraction requires an energy-intensive electrolysis process [3]. When it reacts with water, it produces hydrogen gas (H_2_) and aluminum hydroxide [4,5,6,7,8,9]. Magnesium (Mg) is an alkaline earth metal with light weight, resistance to corrosion, and high strength when alloyed [10,11]. Mg produces hydrogen gas efficiently through reaction with water, yielding H_2_ and magnesium hydroxide [12,13]. Both metals have the characteristic of quickly forming a protective layer of oxide on their surfaces upon contact with oxygen in the air, which protects against corrosion [14,15,16,17,18]. If this layer is removed, it will immediately regenerate if the metal is outdoors. Furthermore, Al crystallizes in an FCC structure and melts at 660.32 °C [19], while Mg crystallizes in an HCP lattice and melts at 651 °C [20]. Al–Mg alloys are thermodynamically susceptible to undergoing corrosion in aqueous solutions and release hydrogen, hydrolyze in water to produce metal hydroxides/oxides and H_2_ as a potential renewable hydrogen source [21,22,23,24,25]. The reaction is faster at higher temperatures, in seawater, or when the passivating oxide layer is disrupted. While Mg acts as an activator in some alloys, it can also form passive layers that slow down the reaction in others [26,27,28,29,30,31]. For these reasons, the Al–Mg alloys are of significant interest. The equilibrium solid phases of the Al–Mg system, the maximum solubility of Mg in Al is 18.9 at. % at 450 °C (eutectic temperature) and the maximum solubility of Al in Mg is 11.8 at. % at 437 °C [32,33,34]. Furthermore, the liquidus, solidus, solvus and solubility lines of the Al–Mg system have been widely studied, which present intermetallic compounds or phases; mainly, the Al_3_Mg_2_ solid solution under 450 °C, commonly named β–phase, has an FCC crystal structure, and the γ–phase (Al_12_Mg_17_) at 58.6 at. % crystallizes as a variant of the cubic α−Mn type [35,36]. These Al–Mg alloys are prone to intergranular corrosion (IGC) due to the formation of the β–intermetallic phase (Al_3_Mg_2_) at grain boundaries [37,38,39]. When Mg content exceeds 3 wt.%, they become susceptible to precipitation of the β−phase along grain boundaries and IGC in seawater, where the grain boundary dissolves preferentially, sometimes leading to exfoliation [29,40,41]. The β−phase and its variants generate stable, equilibrium precipitate plates, particularly at grain boundaries, which results in a decrease in mechanical strength and greater susceptibility to IGC after heat treatment [42,43,44]. However, the precipitation of Mg−rich phases, primarily the β–phase, in Al–Mg alloys is followed by microgalvanic corrosion and dealloying of Mg at grain boundaries, causing IGC [45,46,47].

On the other hand, the hydrolysis of aluminum is denoted by

2Al + 6H_2_O_(*l*)_ → 2Al(OH)_3(*s*)_ + 3H_2(*g*)_ ΔH ≈ –1271.6 kJ/mol (1)

as a potent hydrogen source (1245 mL H_2_/g of Al) releasing ≈ –4.3 kWh per kg of Al [26,48]. Aluminum hydroxides formed, such as bayerite, α−Al(OH)_3_. Another pathway is:

2Al_(*s*)_ + 4H_2_O_(*l*)_ → 2AlO(OH)_(*s*)_ + 3H_2(*g*)_, (2)

which forms aluminum oxide hydroxide (bohemite), depending directly on the temperature [49,50]. Eventually, bayerite and bohemite tend to be precursors of alumina [51].

2Al_(*s*)_ + 3H_2_O_(*l*)_ → Al_2_O_3(*s*)_ + 3H_2(*g*)_. (3)

Similarly, the hydrogen generation from Mg has been extensively studied recently [52,53,54,55,56,57,58,59]. Mg reacts with water via [60]:

Mg_(*s*)_ + 2H_2_O_(*l*)_ → Mg(OH)_2(*s*)_ + H_2(*g*)_ ΔH ≈ –354 kJ/mol. (4)

Considering that β−phase in Al–Mg alloys, with more than 3 wt.% Mg, is a critical precipitate [44], particularly detrimental due to forming a continuous network along grain boundaries [61,62], in many typical acidic and alkaline conditions, in the case of pH ≤ 10 (anodic nature) β−phase is active regarding the aluminum matrix, preferentially causing its dissolution, which originates IGC and stress corrosion cracking (SCC) [62,63]. Conversely, in alkaline conditions (pH = 13) a polarity reversal occurs by becoming cathodic (noble) related to the aluminum matrix [64]. In addition, the dissolution of magnesium through the dealloying of magnesium ions leads to an increase in pH to produce precipitation of Mg(OH)_2_ [29]. Specifically, the hydrolysis of Al–Mg alloys produces H_2_ by the formation of a microgalvanic corrosion caused by the difference in potential between the Al_3_Mg_2_ phase and the Al matrix [31,65]. β–Al_3_Mg_2_ or γ–Al_12_Mg_17_ intermetallic phases are both stiff and brittle; under mechanical load, cracks start easily and propagate rapidly [66,67,68]. Furthermore, β−precipitates and microstructures act as hydrogen traps, influencing susceptibility to embrittlement in Al−alloys. Therefore, the hydrogen generation rate and its yield could be increased when the composition of Al–Mg alloys is modified [69]. In this sense, Mg further improves the affinity of hydrogen for deposition at the grain boundary [70,71]. Mg hydrolyzes more rapidly than Al at room temperature, since passivation by Mg oxide typically involves a porous and unstable layer, which allows the production of hydrogen through continued corrosion, even at sub-zero temperatures [72,73,74,75]. In contrast, the Al and its alloys do not react readily with the water at room temperature, or even with boiling water, because the Al forms an adherent layer of bayerite, boehmite, or alumina. This robust, dense, and highly stable layer acts as a protective barrier that prevents hydrolysis of aluminum, which results in very effective passivation [74,76,77,78]. Hence, to facilitate the hydrolysis reaction of Al–Mg alloys, it is necessary to remove the alumina hydroxide layers during the process. Consequently, researchers have reported chemical, mechanical, and liquid-metal methods to promote the hydrolysis of Al alloys [79,80,81,82,83,84]. 

Furthermore, the corrosion-induced degradation of Al and Mg alloys is primarily driven by Mg segregation at grain boundaries and adjacent regions, facilitating the training of intermetallic phases that act as anodic or cathodic sites, triggering a local microgalvanic corrosion and hydrogen embrittlement (HE) which weakens the structural integrity of the alloy [61,85,86,87]. Likewise, the presence of the intermetallic phase γ –Al_12_Mg_17_ on Mg–Al alloys, frequently considered a cathodic site which leads micro-galvanic corrosion of the α−Mg matrix, can also act as a physical barrier that delays general corrosion depending on its morphology [36,88,89,90,91]. Consequently, this work hypothesizes that a high Mg content in Al−based alloys (with 20 % Mg by weight) will accelerate the corrosion process due to the formation of anodic precipitates [60,92], because the Mg content in an Al–Mg alloy significantly influences the corrosion performance of the alloy [93]. 

High-magnesium Al–Mg alloys have emerged as promising materials to control corrosion in seawater. However, their electrochemical performance is strongly dependent on the alloy microstructure, particularly the distribution, morphology, and connectivity of the β−Al_3_Mg_2_ intermetallic phase, which can be tailored through heat treatment.

**Figure 1:**
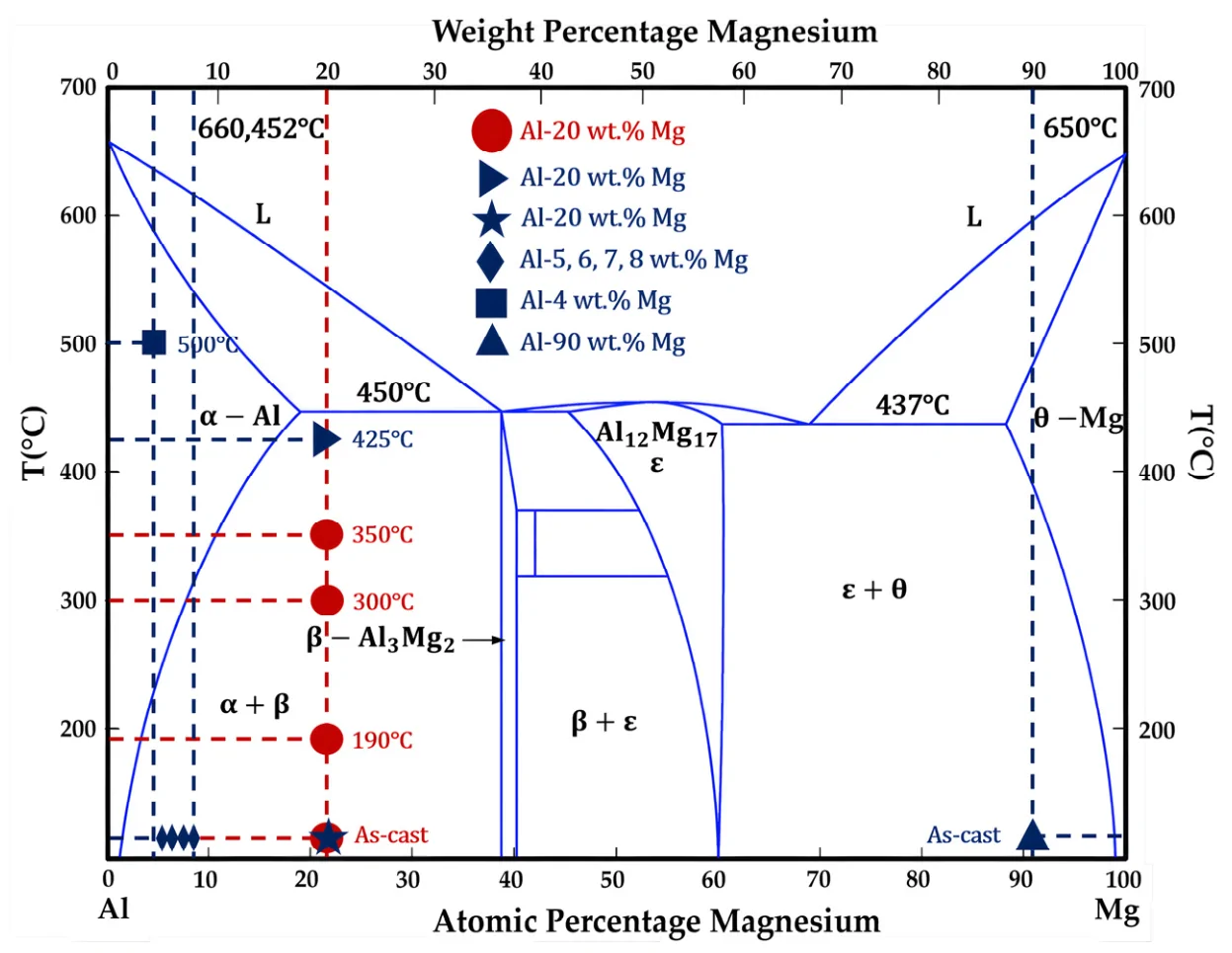
Al–Mg equilibrium phase diagram and heat-treated samples [31,40,92,93,94].

This study proposes a microstructural approach to magnesium redistribution in an Al–20 wt.% Mg alloy via heat treatment (HT) in order to increase susceptibility to corrosion, as summarized in Figure 1. Although it is known that heat treatment can alter the precipitation of β−Al_3_Mg which destabilizes the passive film, this corrosion mechanism remains not fully understood. 

## 2. Materials and Methods

**Figure 2:**
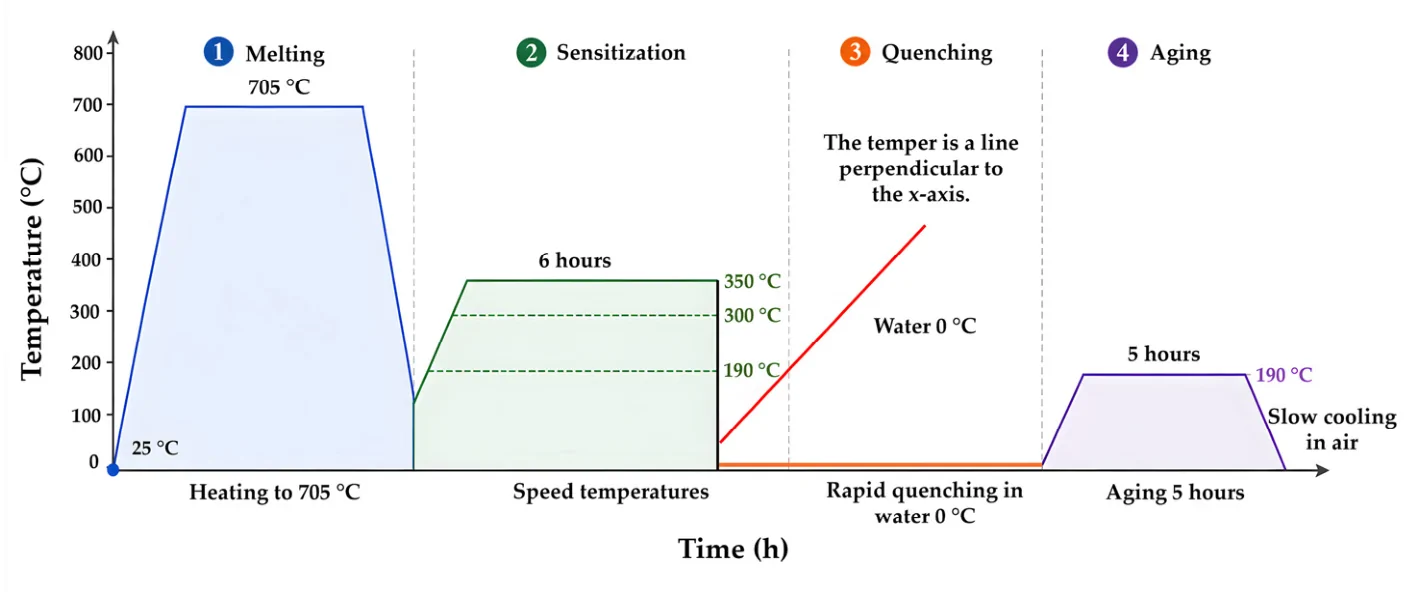
Schematic diagram of processing schedule of the samples.

Binary specimen Al–20 wt.% Mg alloy used in this study was prepared by melting aluminum (99.00% purity) and magnesium (99.99 purity) ingots (Metales Aguila, S.A. de C.V., Mexico). These raw materials were cut into rectangular sections of 15 × 5 × 8 mm. A stoichiometric ratio of 80 wt.% Al and 20 wt.% Mg was melted in a clay–graphite crucible using a resistance furnace (PREFINSA model HR C4/1200, Mexico) at 705 °C, as reported in the literature [95,96]. The molten alloy was then poured into a rectangular steel mold preheated to 200 °C and allowed to cool to room temperature. To modify the microstructure of Al–Mg alloy and promote the formation of the intermetallic phases (mainly Al_3_Mg_2_), the cast Al−20 wt.% Mg ingot was divided into four equal portions; three of them underwent heat treatment (sensitization) for 6 h using a Carbolite CWF 1100 High-Temperature furnace. The first portion was treated at 190 °C (sample HT190), the second at 300 °C (sample HT300), and the third at 350 °C (sample HT350). The fourth portion (as-cast sample) did not undergo any heat treatment (HT) after solidification and was evaluated in its as-cast state. The as-cast specimen was tested in the production state without any subsequent thermal processing (Table 1). Sensitization causes continuous precipitation of the β−phase at grain boundaries, which drastically reduces mechanical strength and increases susceptibility to intergranular corrosion [97]. Then, each of the samples was quenched at 0 °C and subsequently aged at 179 °C for 5 h (Figure 2). 

According to the Mg-rich phase in the Al–Mg equilibrium phase diagram [95], the maximum solid solubility of Mg in Al is 17.4 wt.% at the eutectic temperature of 450 °C, which defines a critical threshold for heat-treating Al alloys with high Mg content, where the α–aluminum solid solution is in equilibrium with the secondary phases involved in this process (β–Al_3_Mg_2_ or γ–Al_12_Mg_17_ intermetallic phases) [35,95]. In the case of cast structures with severe segregation that creates drastic variation in its chemical and mechanical properties (through its intermetallic phases), homogenization implicates maintaining the alloy at temperatures from 190 to 350 °C to modify the composition of these intermetallic phases involved and distribute the Mg uniformly in the Al matrix [97].

For microstructural characterization, the heat-treated (HT) samples were ground using SiC paper (250 to 2000 grit) and polished with 1 μm diamond paste. Subsequently, the samples were chemically etched with a Keller’s reagent (2 mL of HF, 3 mL of HCl, 5 mL of HNO_3_ and 190 mL of water) to reveal the microstructure, grain boundaries, matrix contrast, precipitates, and intermetallic second phases [95]. All chemical reagents were purchased at Fermont, Productos Químicos Monterrey, Mexico). 

The samples were characterized using a JEOL JSM-7600F scanning electron microscope, Japan. Scaning Electron Microscope (SEM) was employed to examine the microstructure, grain boundaries, and secondary phases. In combination with energy-dispersive X-ray spectroscopy (EDS), it was used to qualitatively and quantitatively determine the elemental composition of the microstructure, evaluate the distribution of Mg and Al, and identify elemental segregations and secondary intermetallic phases. An optical microscope, Iroscope, MG-70 model was used to determine the grain size, identify intermetallic phases, and detect microstructural defects. X-ray diffraction (XRD) patterns were acquired using a Bruker D8 Advance, Massachusetts, USA, diffractometer equipped with a Cu-Kα radiation source (λ = 1.5406 Å), operated at an accelerating voltage of 30 kV and a tube current of 30 mA. XRD analysis was performed to identify the crystalline phases present, confirm the formation of solid solutions, and detect the precipitation of secondary intermetallic phases.

The corrosion behavior and mechanism of the samples were determined using various electrochemical analysis techniques. The open-circuit potential (OCP) was monitored continuously for approximately 30 min prior to each electrochemical test, and stabilization was assessed visually, considering the potential as stable when the OCP-time curve reached a steady, flat response with no further significant drift. This qualitative stabilization criterion ensured that the potential remained within a ±2 mV range for a 5-min interval prior to measurements and was consistent with the approach used in previous studies on aluminum-based alloys in chloride-containing media [98], and follows the same experimental protocol previously applied by our group to the Al−20Mg alloy in synthetic seawater [40]. 

Potentiodynamic polarization curves were recorded at a scan rate of 10 mV/min, ranging from –300 mV to +600 mV vs. OCP, to evaluate the susceptibility to degradation and the active–passive behavior in seawater. The synthetic seawater solution (electrolyte) is a reproducible mixture of pure water and dissolved minerals, designed to simulate the chemical composition, salinity, and pH of ocean seawater; it was prepared following the synthetic seawater procedure described in the normative (ASTM-D1141) [99] using reagents of analytical grade. The chemical composition (Table 2) of the synthetic seawater solution is according to [99].

Additionally, electrochemical impedance spectroscopy (EIS) was performed with a 10 mV amplitude over a frequency range of 20 kHz to 0.1 Hz to characterize the surface behavior of the alloy. The EIS data were fitted to the proposed equivalent electrical circuits (EEC) using ZSimpWin 3.0 software. The goodness-of-fit was quantitatively validated via Chi-squared (χ^2^) analysis. 

The corrosion characteristics and mechanism of the samples were determined using various electrochemical analysis techniques in a standard three-electrode cell configuration Hach, XR110 model. Samples of the Al–20 wt.% Mg alloy were embedded in epoxy resin, leaving a controlled exposed geometric work area of 1.0 cm^2^ to act as the working electrode. A saturated calomel electrode (SCE) was used as the reference electrode, and a graphite rod as the counter electrode.

All tests were conducted using a Gill AC potentiostat/galvanostat (ACM Instruments, Cumbria, UK) controlled by a computer.

## 3. Results

### 3.1. Characterization Structural for Heat-Treated Al–20 Wt.% Mg Alloy

**Figure 3:**
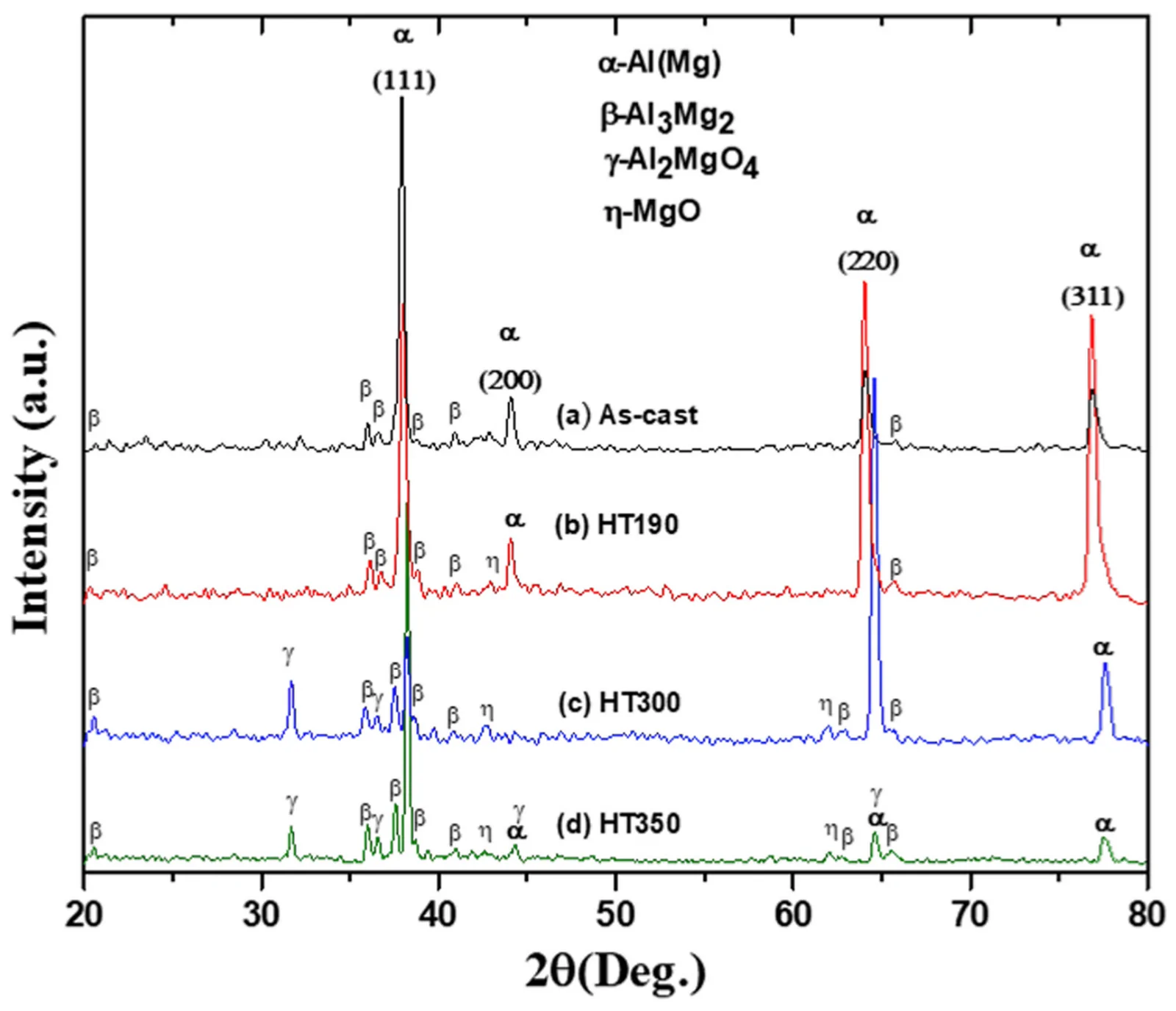
XRD patterns of the Al–20 wt.% Mg alloy in the as-cast condition and after heat treatment for 6 h at different temperatures: (**a**) As-cast, (**b**) HT190, (**c**) HT300, and (**d**) HT350.

Figure 3 shows the X-ray diffraction (XRD) patterns of the alloy in the as-cast condition and after heat treatments at 190, 300, and 350 °C. Based on the Al–Mg binary phase diagram [99], the diffractogram of the as-cast alloy (Figure 3a) indicates the presence of two predominant phases: the α−Al(Mg) solid solution, consisting of Mg dissolved in an Al−rich matrix with a face-centered cubic (FCC) crystal structure, and the β−Al_3_Mg_2_ intermetallic phase. The α−Al(Mg) phase was identified according to the ICDD PDF No. 04-0787, corresponding to a cubic structure with space group Fm-3m (No. 225) and a lattice parameter of a = 4.04994 Å. The β−Al_3_Mg_2_ intermetallic phase was identified according to the ICDD PDF No. 29-0048, with a cubic structure, space group Fd-3m (No. 227), and a lattice parameter of a = 28.239 Å [100,101]. The coexistence of these phases is consistent with the alloy composition and the expected solidification behavior of high-magnesium Al–Mg alloys.

The β–Al_3_Mg_2_ intermetallic phase was identified by a series of overlapping diffraction peaks concentrated primarily within the 35–42° 2*θ* region, where its most intense reflections are located. Although the formation of β–Al_3_Mg_2_ contributes to precipitation strengthening and enhances the mechanical performance of the alloy, excessive segregation of this phase at grain boundaries is known to reduce corrosion resistance by facilitating localized galvanic interactions [93].

As shown in Figure 3b–d, the diffraction peaks associated with the β−Al_3_Mg_2_ phase become progressively narrower and more intense as the heat-treatment temperature increases, suggesting an increase in the crystallinity and/or development of the β−Al_3_Mg_2_ phase [44,100]. In addition, the α−Al(Mg) reflections in the HT190, HT300, and HT350 samples exhibit changes in their relative intensities, particularly for the (200), (220), and (311) planes, suggesting the development of a preferential crystallographic orientation during heat treatment and subsequent quenching. A slight shift in the α−Al(Mg) reflections toward higher 2θ angles is also observed, indicating a reduction in the lattice parameter of the α−Al(Mg) solid solution. This behavior can be attributed primarily to the diffusion of Mg atoms from the supersaturated α−Al(Mg) matrix and their subsequent incorporation into β−Al_3_Mg_2_ precipitates. The resulting depletion of Mg from the α−Al(Mg) solid solution reduces the lattice parameter of the Al−rich FCC matrix, producing lattice contraction and a corresponding shift in the diffraction peaks toward higher 2θ angles, in accordance with Bragg’s law. The chemical reaction that can explain the formation of the products identified in Figure 3 is the following:

2Al(*s*) + 6Al_3_Mg_2_(*s*) + 21O_2_(*g*) → 10Al_2_MgO_4_(*s*) + 2MgO(*s*) (5)

The elevated treatment temperatures employed for the HT300 and HT350 conditions enhanced magnesium diffusion and redistribution, promoting a more homogeneous precipitation of the β–Al_3_Mg_2_ intermetallic phase throughout the alloy matrix. Furthermore, rapid quenching retained a fraction of Mg in supersaturated solid solution while suppressing the premature formation of equilibrium precipitates [24,102,103,104,105,106,107]. Such microstructural modifications are commonly associated with increased hardness and strength, although they may also promote more brittle fracture behavior and increase susceptibility to localized corrosion.

In addition to the α−Al(Mg) and β−Al_3_Mg_2_ phases observed in Figure 3b–d, diffraction peaks corresponding to MgO were identified in the HT190–HT350 samples. The MgO phase was identified according to ICDD PDF No. 89-7746 and is characterized by a cubic rock-salt-type structure with space group Fm-3m (No. 225) and a lattice parameter of a = 4.2198 Å. Likewise, diffraction peaks corresponding to MgAl_2_O_4_, commonly known as the spinel phase, were detected in the HT300 and HT350 samples. The MgAl_2_O_4_ spinel phase was identified by comparison with the crystallographic structure reported in the American Mineralogist Crystal Structure Database (AMCSD), entry 99-100-2939, corresponding to a cubic structure with space group Fd-3m (No. 227) and a lattice parameter of a = 7.9707 Å.

The formation of these oxide phases is attributed to oxidation processes occurring during exposure to elevated temperatures in air. The appearance of MgAl_2_O_4_ at higher heat-treatment temperatures is consistent with enhanced Mg outward diffusion and preferential oxidation at the alloy surface, processes that become increasingly significant with increasing temperature. The detection of MgAl_2_O_4_ spinel at 300 and 350 °C indicates the formation of a thermodynamically stable Mg–Al mixed oxide under the applied thermal conditions.

**Figure 4:**
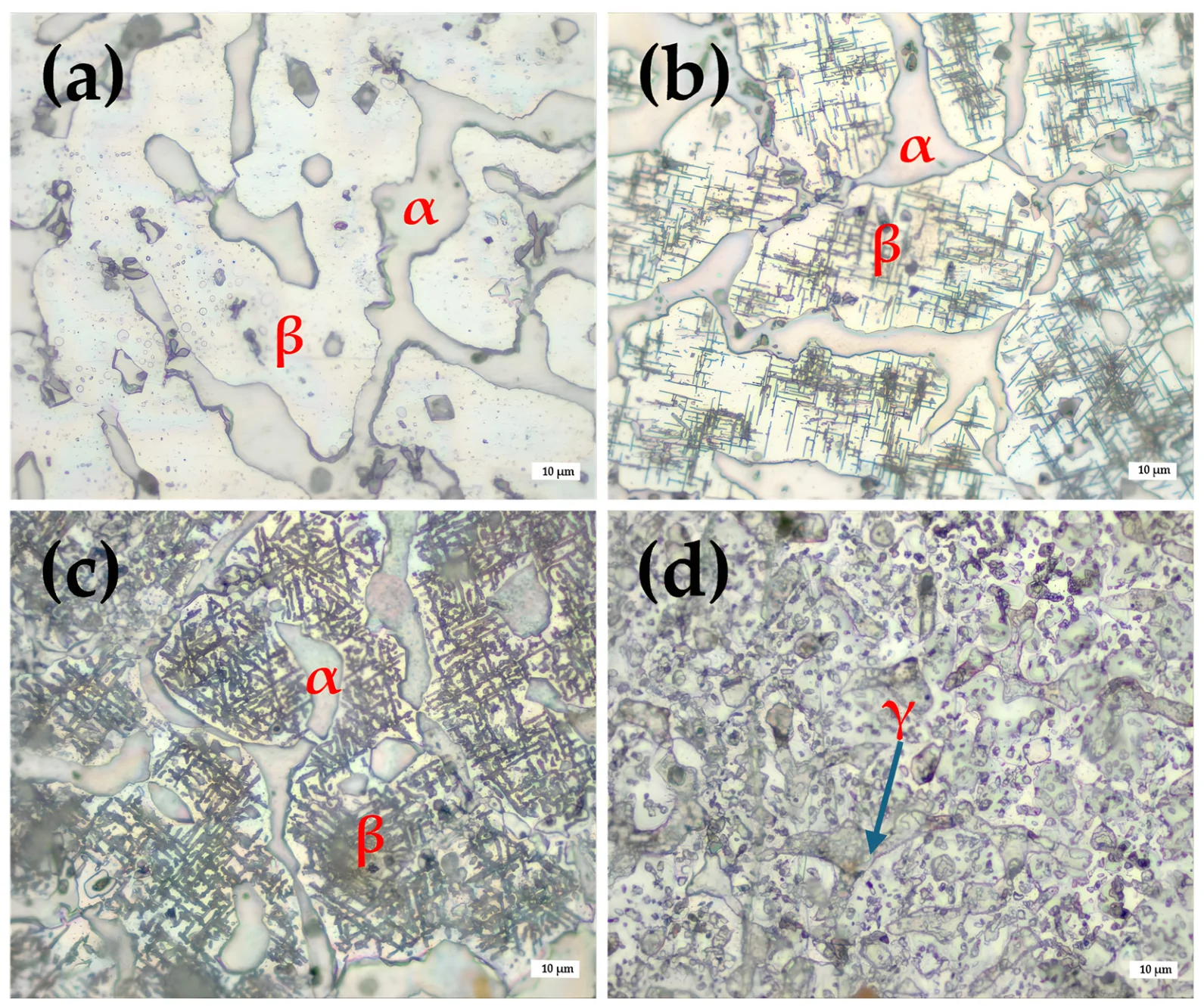
OM micrographs of the specimens in unexposed condition: (**a**) As-cast, (**b**) 190 °C, (**c**) 300 °C, and (**d**) 350 °C.

Figure 4 presents optical microscopy (OM) of the samples before and after exposure to synthetic seawater. In Figure 4a, corresponding to the as-cast sample, the typical α−Al(Mg) and β−Al_3_Mg_2_ phases are observed, as identified by the regions marked in the figure. The as-cast condition exhibited early microstructural evidence of β−Al_3_Mg_2_ intermetallic phase nucleation, likely associated with Mg segregation during solidification. The intermetallic regions appeared preferentially localized, while the surrounding α−Al(Mg) matrix remained relatively clean and uniform. Such behavior suggests that the β phase initially develops through localized compositional fluctuations prior to extensive intergranular growth during subsequent thermal exposure. 

Heat treatment at 190 °C (Figure 4b) followed by rapid quenching promoted the initial microstructural evolution of the β−Al_3_Mg_2_ intermetallic phase, characterized by the appearance of elongated growth features within the intermetallic regions. This behavior is consistent with diffusion-controlled growth under moderate thermal activation. Concurrently, the α−Al(Mg) matrix surrounding these regions exhibited localized solute depletion, indicating possible Mg redistribution from the supersaturated matrix toward the β phase during thermal exposure. 

Figure 4c shows that heat treatment at 300 °C promoted a substantial increase in the density of elongated structures corresponding to the β−Al_3_Mg_2_ intermetallic phase. The intermetallic regions became more extensive and interconnected relative to the sample treated at 190 °C, reflecting accelerated diffusion kinetics activated at this temperature. In parallel, the α−Al(Mg) matrix in the surrounding regions exhibited progressive solute depletion, indicating continued Mg redistribution toward the growing intermetallic phase and the gradual transformation of the microstructure during thermal exposure. 

The alloy treated at 350 °C and ice-water-quenched (Figure 4d) displayed pronounced microstructural modifications within the β−Al_3_Mg_2_ intermetallic regions. Unlike the elongated morphology observed at lower treatment temperatures, the β phase was fragmented into finer and more discrete particles, homogeneously distributed throughout the intermetallic network. This transformation indicates that the increased thermal energy enhanced the instability and breakdown of the elongated β−Al_3_Mg_2_ structures. Additionally, the α−Al(Mg) matrix contained a dispersed population of fine particles, identified as γ−MgAl_2_O_4_ spinel (marked by an arrow in Figure 4). Their presence is attributed to oxidation reactions occurring during exposure to elevated temperatures in an air atmosphere, while the subsequent rapid quenching preserved these oxidation products and inhibited further microstructural coarsening.

To further quantify the microstructural evolution of the intermetallic phase, Table 3 summarizes the approximate area fraction of the β−Al_3_Mg_2_ phase, determined from three metallographic images for each condition using ImageJ 1.54g software. The quantitative metallographic analysis revealed a progressive increase in the β−Al_3_Mg_2_ area fraction from the as-cast condition to HT300, followed by a decrease at HT350. The as-cast alloy exhibited an area fraction of 14.343 ± 1.054%, which increased to 33.674 ± 2.608% after heat treatment at 190 °C. The highest β−Al_3_Mg_2_ area fraction was obtained at 300 °C, reaching 47.411 ± 2.881%. At 350 °C, the area fraction decreased to 35.546 ± 2.100%. These results indicate that HT300 promotes the highest measured area fraction of β−Al_3_Mg_2_, whereas a further increase in temperature to 350 °C results in a reduction in the measured intermetallic area fraction. The decrease in the β−Al_3_Mg_2_ area fraction observed in the HT350 condition may be associated with the formation and growth of oxide phases, particularly magnesium aluminate spinel (MgAl_2_O_4_).

The increased precipitation of β–Al_3_Mg_2_, the depletion of Mg from the α–Al(Mg) matrix, and the formation of heterogeneous oxide phases can generate localized galvanic couples and microstructural discontinuities that facilitate anodic dissolution. Consequently, the HT300 and HT350 samples are expected to exhibit enhanced corrosion susceptibility when exposed to synthetic seawater, promoting the breakdown of the native passive film and accelerating aluminum–water reactions.

### 3.2. Effect of Heat Treatment on Corrosion Behavior of Al–20 Wt.% Mg Alloy

The open-circuit potential (OCP) values for the Al–Mg specimens are summarized in Table 4. In terms of thermodynamic electrochemical activity, the alloys exhibited a trend in the order HT350 > as-cast > HT190 > HT300, suggesting that the precipitation of the β–phase significantly changes the OCP. As reported by Ikeuba et al. [64], secondary phases are main drivers of susceptibility to corrosion in Al alloys. Specifically, the morphology, spatial distribution, and chemical composition of the intermetallic precipitates are decisive factors that govern the overall electrochemical response of the matrix.

**Figure 5:**
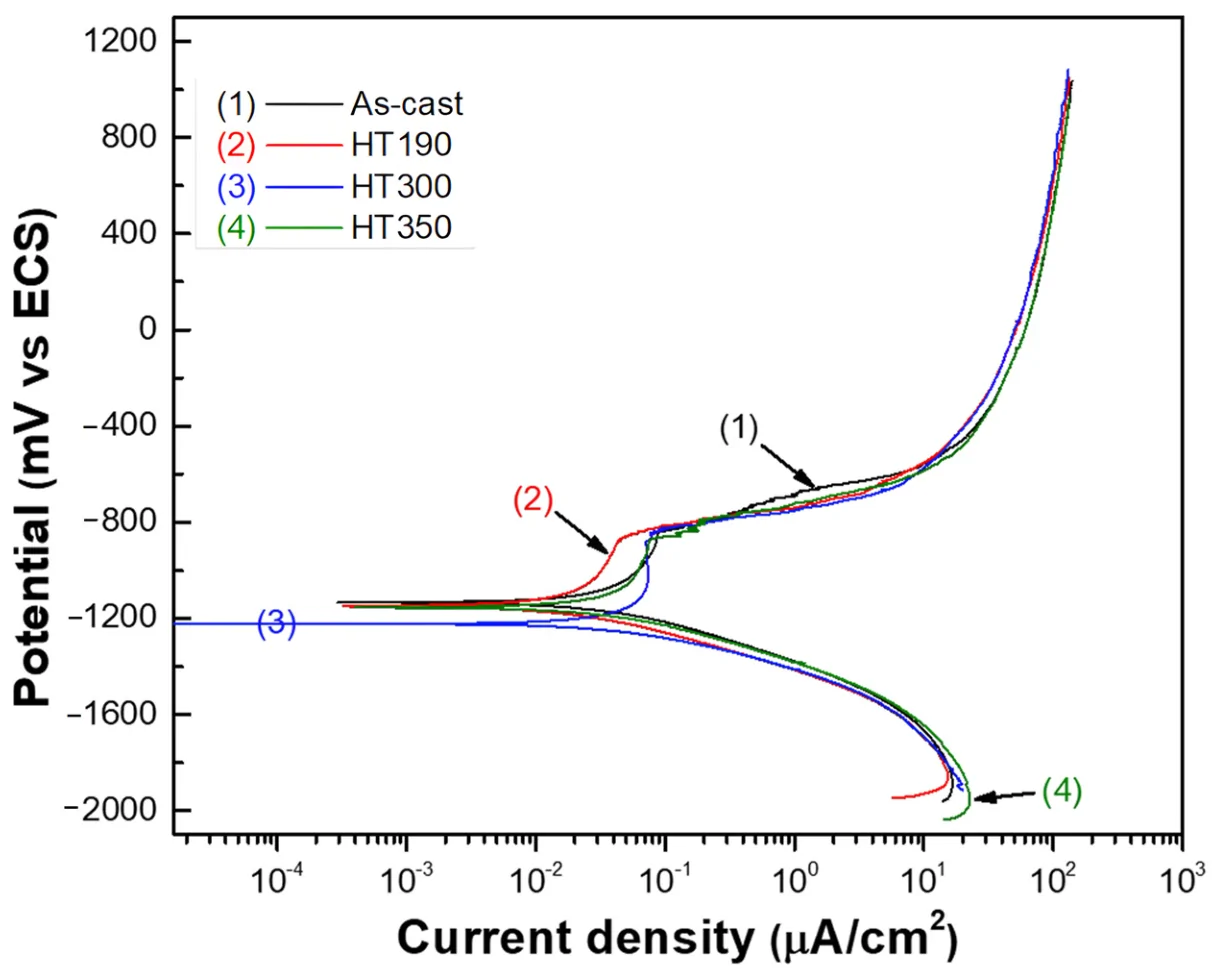
Potentiodynamic polarization curves for as-cast and heat-treated Al-20 wt.% Mg alloys in synthetic seawater.

Figure 5 illustrates the potentiodynamic polarization curves behavior of the Al–20 wt.% Mg alloy in its as-cast and heat-treated (190, 300, and 350 °C) states in synthetic seawater (pH 8.2). The electrolytic response is primarily characterized by charge-transfer-controlled kinetics, accompanied by a transient tendency for pseudo-passivation. Notably, the HT350 °C alloy exhibits a shift in corrosion potential and an increase in corrosion current density (*i_corr_*) relative to the as-cast condition. This acceleration in the corrosion rate [12,25,56] suggests potential conditions for H_2_ evolution via the cathodic reaction. Although this aligns with the reported electrochemical kinetics, hydrogen generation was not directly quantified. The corresponding electrochemical kinetic parameters are detailed in Table 4. 

The differences in corrosion current density among the evaluated conditions are supported by convergent evidence from multiple independent electrochemical parameters, rather than resting on the icorr values in isolation. The variation in icorr with heat-treatment temperature (Table 3) is accompanied by a systematic, physically interpretable shift in the anodic and cathodic Tafel slopes (βa, βc), consistent with the transition in passive-film stability. This trend is also consistent with the electrochemical behavior previously reported for the same Al−20 wt.% Mg alloy system in synthetic seawater [99].

The aging heat treatment at 350 °C significantly accelerated the corrosion rate of the Al−20 wt.% Mg alloy. The observed shifts in the anodic Tafel constants (βa) suggest a transition in the electrochemical kinetic mechanisms across the various metallurgical states. This behavior is governed by the precipitation of corrosion products, whose accumulation changes the overall dissolution kinetics. The measured anodic slopes (46.91, 53.86, and 47.09 mV/decade) confirm that the anodic process deviates from pure charge-transfer control, indicating a complex interplay between metallic dissolution and the transient stability of the native passive film. While the values near 47 mV/decade suggest a consistent mechanistic path, the increase to 53.86 mV/decade points toward a more dominant role of the surface oxide/hydroxide film in limiting the anodic current.

The pitting potentials (Epit), obtained from the potentiodynamic curves, are summarized in Table 4. Epit values ranged narrowly between −831.3 mV (as-cast) and −851.7 mV (HT300), with HT190 and HT350 falling within this same interval (−839.8 mV and −836.9 mV, respectively). This relatively narrow range (~20 mV) indicates that the onset of pitting is governed by a common depassivation mechanism across all metallurgical conditions, associated with chloride attack at β−Al_3_Mg_2_/α−Al(Mg) interfaces. Unlike icorr and Rct, which follow a clear monotonic trend with increasing heat-treatment temperature, Epit does not vary systematically with the degree of β−phase development, suggesting that the pitting-initiation potential and the overall corrosion rate are controlled by partially distinct microstructural factors within the studied temperature range. 

According to Zhou et al. [29], an increase in Mg content coupled with a reduction in Cr leads to higher passive current densities and increased pit depths, alongside a decrease in both polarization resistance (*Rp*) and the Al_2_O_3_/Al(OH)_3_ ratio in the corrosion product layer. The *Rp* values, calculated via the linear polarization region (±20 mV vs. *E_corr_*), are presented in Table 4. The as-cast Al−20 wt.% Mg alloy displayed an *R_p_* of 400 Ω·cm^2^, which significantly dropped to 285.71 Ω·cm^2^ for the HT 350 °C condition. This trend confirms the higher electrochemical reactivity of the heat-treated alloy, where a lower *Rp* correlates with an accelerated corrosion rate, in agreement with the findings of Flores-Chan [40].

**Figure 6:**
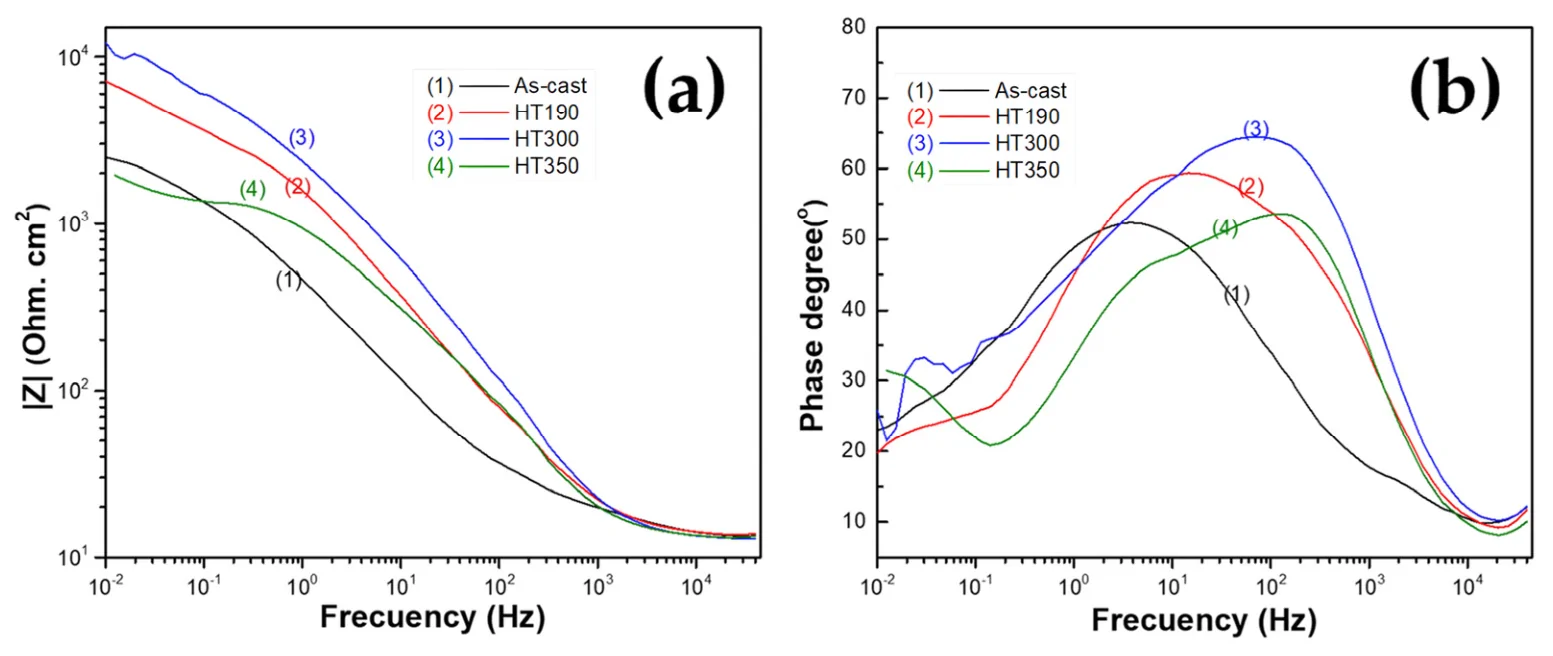
Electrochemical impedance spectroscopy for all samples: (**a**) Bode magnitude (modulus), and (**b**) Bode phase angle plots.

For the specimen heat-treated at 350 °C, the Al_2_O_3_ passive film exhibited highly unstable behavior in the aggressive synthetic seawater medium. For the specimen heat treated at 350 °C, the Al_2_O_3_ passive film exhibited highly unstable behavior in the aggressive synthetic seawater (SS) medium. The resulting increase in the corrosion rate was manifested by localized cracking and the accumulation of corrosion products, indicating enhanced disruption of the passive film. This corrosion behavior may favor aluminum–water interactions and suggests that the HT350 condition warrants further investigation for its potential application in hydrogen generation [58,59]. The Bode plots in Figure 6a reveal a resistive response at high frequencies (103–104 Hz), while a capacitive contribution dominates the 102–103 Hz range [109,110]. This transition underscores the profound impact of the thermal cycle on the alloy’s electrochemical signal. Particularly, the phase angle remains below 10° within the 40 kHz to 1 Hz interval, identifying a dissolution process under charge-transfer control (activation), where the overall rate is dictated by the interface resistance between the Al–Mg matrix and the electrolyte.

Figure 6b illustrates the evolution of the phase angle (*θ*), reflecting the morphological transitions and the development of corrosion product layers on the Al−20 wt.% Mg alloy [61,62]. The as-cast specimen exhibited a peak phase angle of 64°, representing the capacitive contribution of the electrochemical double layer. Conversely, the heat-treated samples (190 °C and 350 °C) displayed lower maximum phase angles of 59° and 53° within the 100–102 Hz and 101–103 Hz frequency domains, respectively.

**Figure 7:**
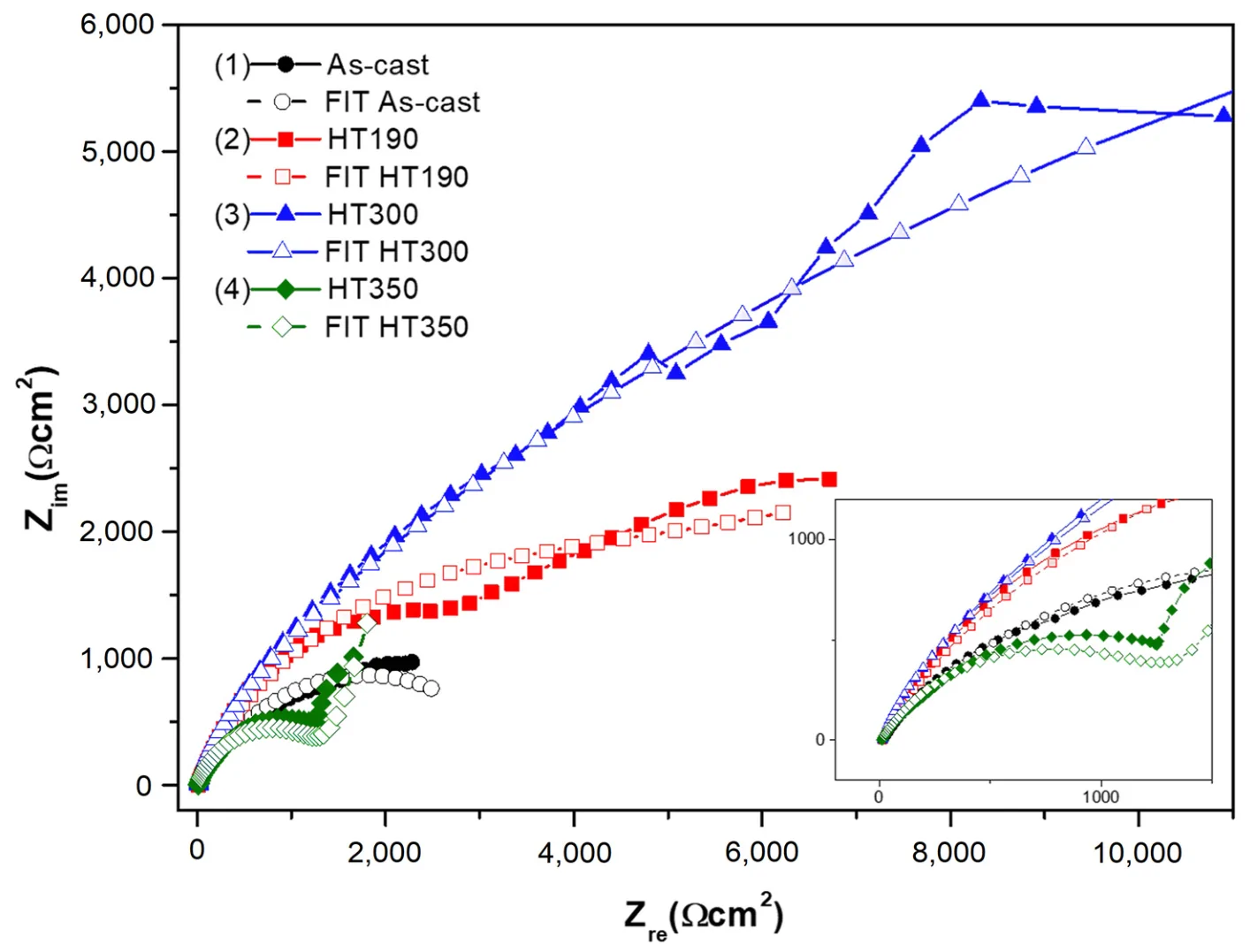
Nyquist plots of the Al−20 wt.% Mg alloy under different heat-treatment conditions.

This reduction in the phase angle magnitude suggests a more resistive or pseudo-capacitive response, likely due to the increased heterogeneity of the surface and the formation of a less protective native passive film at higher aging temperature. The influence of thermal history on the stability of the Al_2_O_3_ film was evaluated via EIS, with the resulting Nyquist plots presented in Figure 7. Following the established relationship where charge-transfer resistance (Rct) is inversely proportional to the corrosion current density [8,64] the Rct values decreased in the order HT190 °C > as-cast > HT300 °C > HT350 °C, with the HT350 condition exhibiting the lowest charge-transfer resistance and, consequently, the highest electrochemical reactivity. This hierarchy underscores the role of precipitate-matrix coherency and its impact on hydrogen trapping mechanisms [69,76]. The specimen aged at 350 °C exhibited the highest susceptibility to pitting, a phenomenon driven by the microgalvanic coupling between the Mg-rich precipitates and the depleted Al matrix [8].

**Figure 8:**
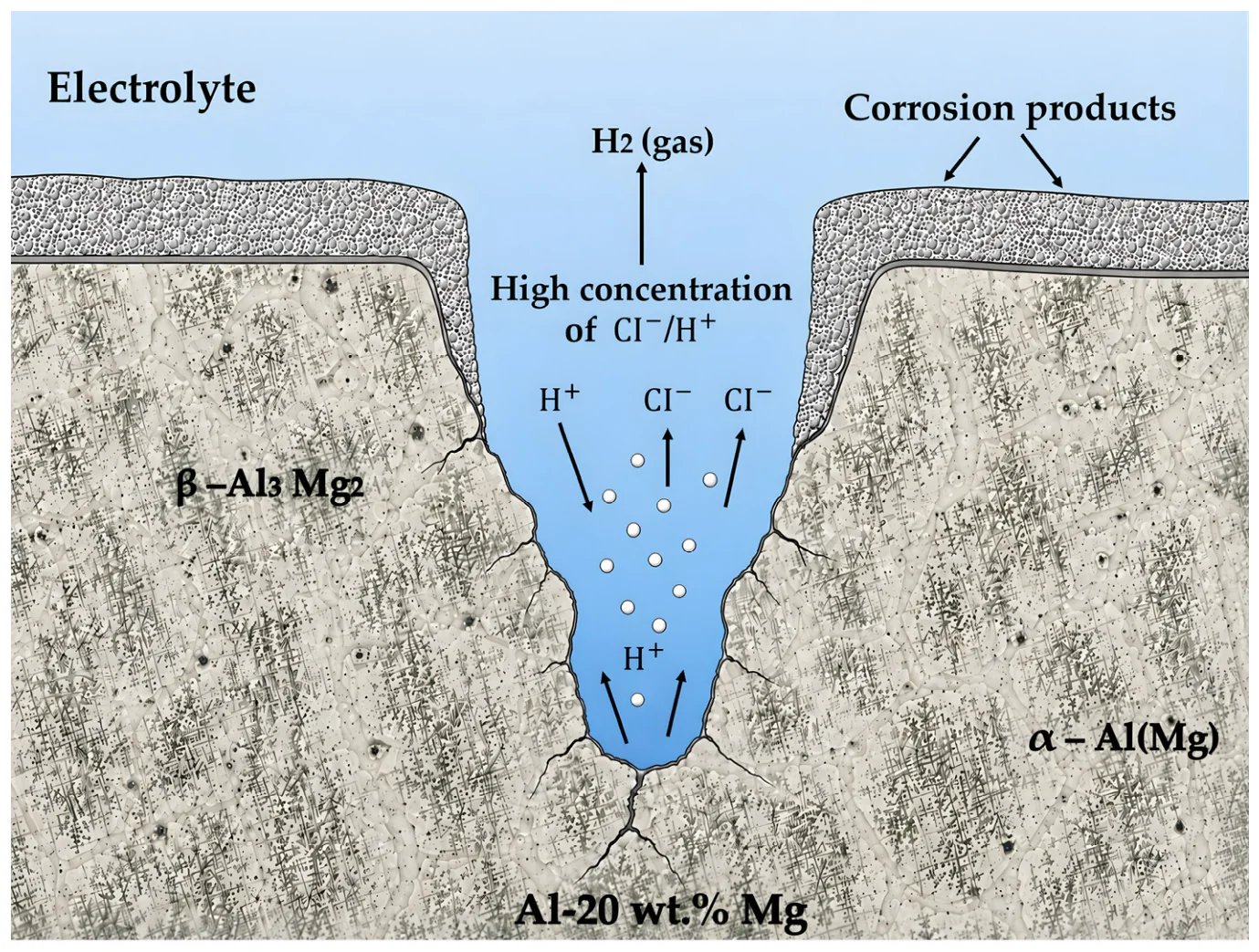
The physical scheme of the corrosion of Al−20 wt.% Mg alloy in SS.

Dissolution under the different metallurgical samples, as-cast, HT190, HT300, and HT350, is a direct consequence of this microstructural heterogeneity, which compromises the integrity of the native passive film. To further elucidate the interaction between corrosion products and the electrochemical response, a physical scheme of the interface is provided in Figure 8, adapted from Liu et al. [111].

**Figure 9:**
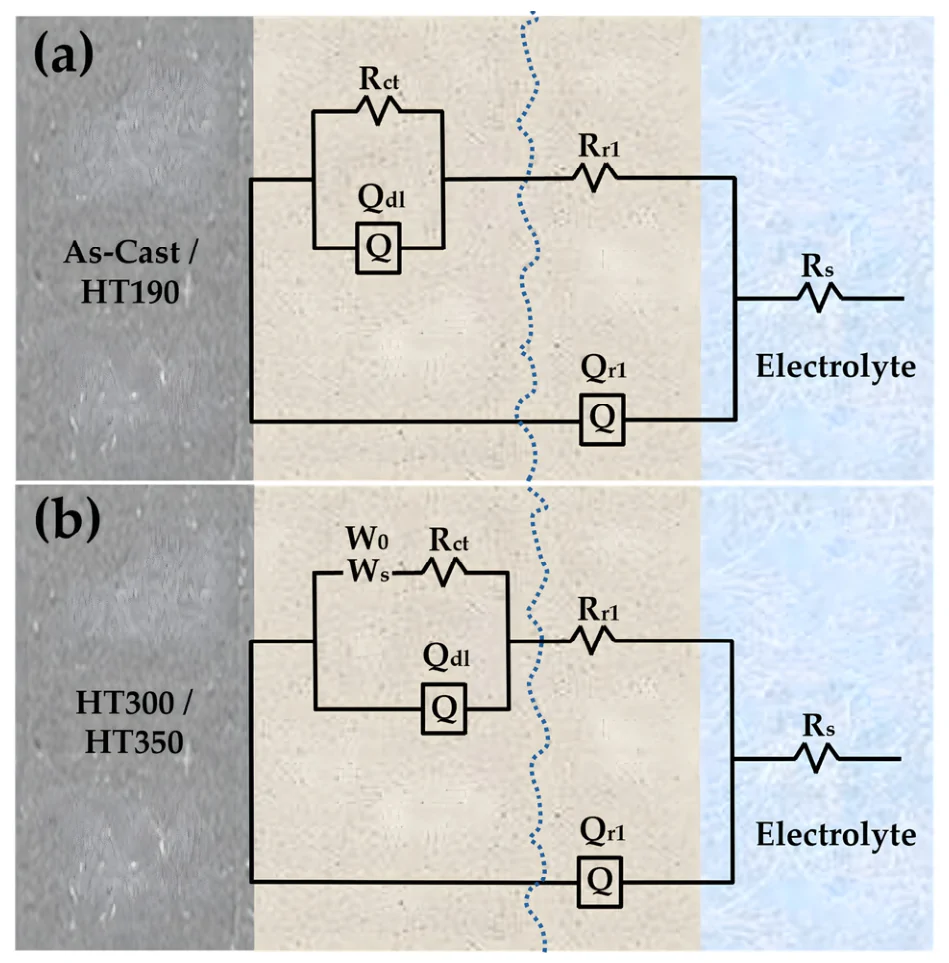
Equivalent circuit model proposed for the electrochemical impedance for the Al-20 wt.% Mg alloy: (**a**) Heat-treated 300 °C and 350 °C; (**b**) in the as-cast and heat-treated 190 °C [111].

Building upon the physical scheme presented in Figure 8, the equivalent electrical circuit (EEC) illustrated in Figure 9 was developed to simulate the electrochemical response. The correlation between the experimental data (symbols) and the model-derived fits (lines) is depicted in Figure 9. To evaluate the goodness-of-fitness and ensure no statistically significant deviation exists between the observed and predicted impedance values, a Chi-squared (χ^2^) analysis was performed; the resulting coefficients are detailed in Table 5.

The equivalent electrical circuit illustrated in Figure 9a,b incorporates Rs as the solution resistance. The first time constant, consisting of a constant phase element (Qrl) in parallel with a resistance (Rrl), models the capacitive and resistive behavior of the corrosion product layer. The second time constant (Qdl/Rct) characterizes the charge-transfer kinetics across the electrical double layer. As noted by Liu, Y., et al. (2022), the corrosion susceptibility of these alloys is driven by a high-volume fraction of matrix precipitates and the morphological evolution of grain boundaries, specifically the discontinuity of precipitates and the development of a precipitate-free zone (PFZ), which act as a preferential site for localized attack [112,113].

The model in Figure 9a by HT300 and HT350 retains the same two RC-type time constants described above but introduces an additional Warburg element (W) in series within the low-frequency branch. The Warburg impedance accounts for semi-infinite linear diffusion of electroactive species (Mg^2+^, or corrosion-reaction products through the thickened product layer formed at higher heat-treatment temperatures). Physically, this reflects a transition from a purely activation-controlled process to a mixed activation–diffusion-controlled mechanism, consistent with a coarser, less permeable surface film and with the metallurgical evolution of the alloy (grain-boundary precipitate coarsening and precipitate-free zone widening) at 300–350 °C.

The Al−20 wt.% Mg alloy across all metallurgical conditions (as−cast and HT at 190, 300, and 350 °C) undergoes spontaneous passivation, forming a natural oxide film upon atmospheric exposure. This phenomenon is profoundly modulated by the microstructural changes induced during heat treatment. According to [114], enhanced resistance to hydrogen embrittlement can be attained by tailoring the distribution of soluble secondary phases, which effectively reduces the hydrogen concentration trapped at dislocations. However, the subsequent formation of non-adherent and porous corrosion products signifies a continuous degradation of the metallic substrate, compromising the integrity of the native passive layer. 

The variation in i_corr_ values derived from Tafel extrapolation is numerically narrow; the electrochemical activation of the heat-treated alloy is unequivocally validated by the robust convergence of independent DC and AC parameters. The polarization resistance (Rp), measured in the linear polarization domain, demonstrated a nearly four-fold reduction from 1111.11 Ω∙cm^2^ in the most resistant state (HT190) to 285.71 Ω*cm^2^ in the most active state (HT350). This dramatic decrease in DC resistance is perfectly mirrored by the non-destructive AC measurements, where the charge-transfer resistance (Rct) dropped from 4455 Ω∙cm^2^ to 1115 Ω∙cm^2^. The strong correlation between Rp and the rigorously fitted Rct (supported by χ^2^ approx. 10^−3^) confirms that the continuous β−phase network developed at 350 °C fundamentally destabilizes the passive film and accelerates interfacial charge-transfer kinetics. The substitution of ideal capacitors with Constant Phase Elements (CPE) in the equivalent electrical circuits accurately models the frequency dispersion arising from this extensive microstructural heterogeneity and localized dissolution at the α/β interfaces. Furthermore, the inclusion of the Warburg impedance (W) for the HT300 and HT350 conditions successfully captures the mass-transport diffusion limitations imposed by the thickened, porous corrosion product layers (such as Mg_4_Al_2_(OH)_14_∙H_2_O and MgO), providing a solid mechanistic basis for the enhanced corrosion susceptibility without relying solely on the statistical variance of icorr.

Therefore, EIS-derived charge-transfer resistance (Rct, Table 4) independently follows the same hierarchy: Rct decreases from 4455 Ω·cm^2^ (HT190) to 3537 Ω·cm^2^ (as−cast) to 2985 Ω·cm^2^ (HT300) to 1115 Ω·cm^2^ (HT350). Where the sample HT350 has the lowest charge-transfer resistance, consequently, the highest electrochemical reactivity is identified, which is consistent with the potentiodynamic polarization results (highest icorr, lowest Rp, Table 3). This convergence between two independent electrochemical techniques (CP and EIS), together with the Tafel parameters, supports the validity of the results and the mechanistic basis for the differences observed in the corrosion current density of the heat-treated sample (HT350).

### 3.3. Structural Characterization of Corrosion Products on the Al–20 Wt.% Mg Alloy

**Figure 10:**
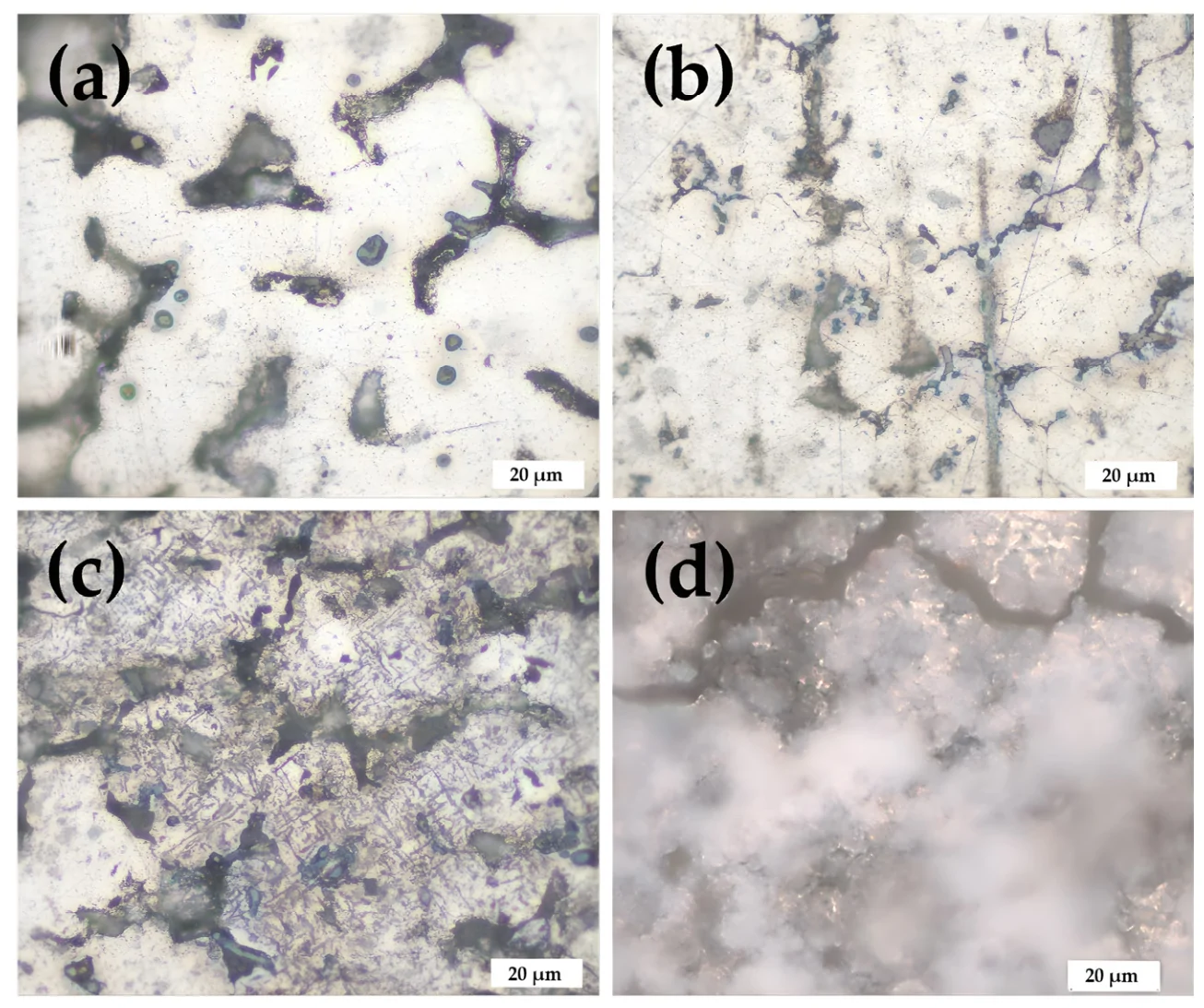
OM micrographs of the corroded surface of Al−20 wt.% Mg alloys in synthetic seawater, after being subjected to heat treatment: (**a**) As−cast, (**b**) 190 °C, (**c**) 300 °C, and (**d**) 350 °C.

Figure 10 presents representative OM micrographs of the as−cast and heat-treated samples after 6 h of corrosion testing in synthetic seawater. The as−cast sample shown in Figure 10a exhibits localized corrosion cavities that are preferentially distributed along the grain boundaries and around fine precipitates within the α−Al(Mg) matrix. The observed morphology indicates the onset of localized corrosion promoted by the heterogeneous microstructure. This behavior is attributed to microgalvanic coupling between the α−Al(Mg) matrix and the β−Al_3_Mg_2_ intermetallic phase, which generates localized electrochemical potential differences that accelerate preferential anodic dissolution of the β−Al_3_Mg_2_ precipitates and the immediately adjacent α−Al(Mg) regions, promoting the subsequent formation of corrosion cavities. The non-uniform distribution of the attack highlights the strong dependence of the corrosion response on the microstructural features of the alloy, particularly the morphology and spatial distribution of the β−Al_3_Mg_2_ phase. Figure 10b shows that the alloy treated at 190 °C displayed an apparent improvement in corrosion resistance during exposure in synthetic seawater. The α−Al(Mg) matrix presented a lower concentration of corrosion cavities compared with the as−cast condition, indicating a reduction in localized anodic dissolution. This behavior may be associated with the initial redistribution and controlled growth of the β−Al_3_Mg_2_ intermetallic phase during thermal exposure, which could have altered the local electrochemical activity and reduced the severity of microgalvanic corrosion processes. The microstructure corresponding to the alloy treated at 300 °C (Figure 10c) revealed a marked deterioration after exposure in synthetic seawater. Extensive cavity formation was observed throughout the α−Al(Mg) matrix, indicating intensified localized corrosion processes relative to the 190 °C condition. Additionally, discrete pitting sites developed within the β−Al_3_Mg_2_ intermetallic phase regions, suggesting that the increased intermetallic growth and connectivity generated microstructural conditions favorable for localized electrochemical instability. This behavior may be associated with enhanced microgalvanic coupling and localized chloride accumulation at the α/β interfaces. Figure 10d shows that the sample heat-treated at 350 °C experienced the most severe corrosion attack after electrochemical exposure in synthetic seawater. The corrosion cavities developed within the α−Al(Mg) matrix became highly interconnected, forming an extensive degradation network across the α−phase regions. This morphology indicates an accelerated localized corrosion process associated with the microstructural transformation and redistribution of the β−Al_3_Mg_2_ intermetallic phase, induced by the heat treatment. Furthermore, a layer of corrosion products is observed covering large areas of the surface, revealing the degradation of the exposed surface of the alloy.

**Figure 11:**
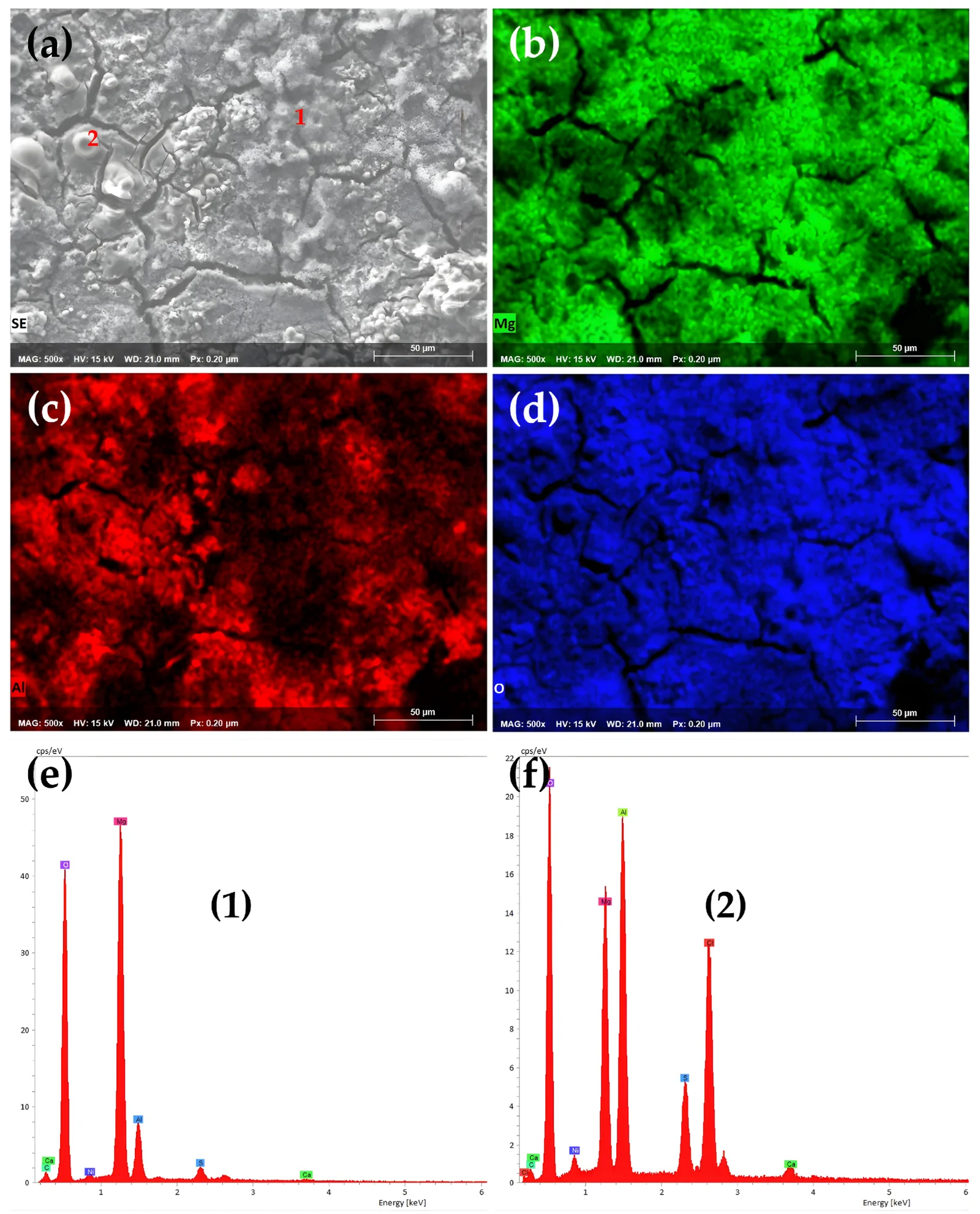
SEM imaging using secondary electrons for sample HT350 (**a**); elemental mapping for Mg (**b**), Al (**c**), and O (**d**); and the EDS spectra of regions 1 (**e**) and 2 (**f**) in (**a**).

The chemical composition of the phases of the corroded sample HT350 was analyzed by secondary electron imaging, elemental mapping, and point energy-dispersive X-ray spectroscopy (EDS) analyses coupled with SEM (Figure 11). Figure 11a shows a detailed view of the interconnected network of cavities and cracks caused by corrosion in synthetic seawater. Elemental mapping for Mg and Al displays a homogeneous distribution throughout the matrix, except in dark areas (corresponding to the β−Al_3_Mg_2_) that reveal degradation caused by corrosion (Figure 11b,c). More dark areas are observed in Figure 11c, indicating a severe corrosion attack. Figure 11d shows the oxygen elemental map, which reveals a widespread distribution of oxygen across the entire analyzed surface. This finding indicates the formation of corrosion products enriched in aluminum and magnesium oxides and/or hydroxides. Figure 11e,f present the EDS spectra acquired from regions (1) and (2) respectively (indicated in Figure 11a). These analyses provide valuable information regarding the chemical composition of the amorphous-like layer covering the alloy surface, which is associated with the corrosion products formed during electrochemical exposure to synthetic seawater. The EDS spectrum obtained from region (1) is characterized by a high concentration of Mg and O, accompanied by a lower Al content. This composition suggests that the amorphous layer is predominantly composed of magnesium-rich corrosion products, likely to include magnesium hydroxide species, together with aluminum-containing hydroxides or oxides. Furthermore, the possible formation of mixed Al–Mg–O–H compounds, such as layered double hydroxides or hydrated mixed corrosion products, cannot be excluded. Small amounts of Ca, S, Cl, and N were also detected, indicating the incorporation of ionic species originating from the synthetic seawater electrolyte into the corrosion product layer. In contrast, the EDS spectrum collected from region (2) reveals a composition dominated by O, Mg, and Al, further supporting the presence of corrosion products based on aluminum and magnesium oxides and hydroxides. However, this region exhibits a higher concentration of electrolyte-derived elements, particularly Ca, Cl, and S, suggesting a greater accumulation or precipitation of salts from the synthetic seawater on the corroded surface. These results indicate that the corrosion layer is morphologically heterogeneous, consisting of a complex mixture of aluminum- and magnesium-based corrosion products together with deposits of ionic species originating from the marine electrolyte. The heterogeneous nature of this layer is consistent with the localized corrosion processes observed around the β−Al_3_Mg_2_ intermetallic phase and the progressive degradation of the α−Al(Mg) matrix.

**Figure 12:**
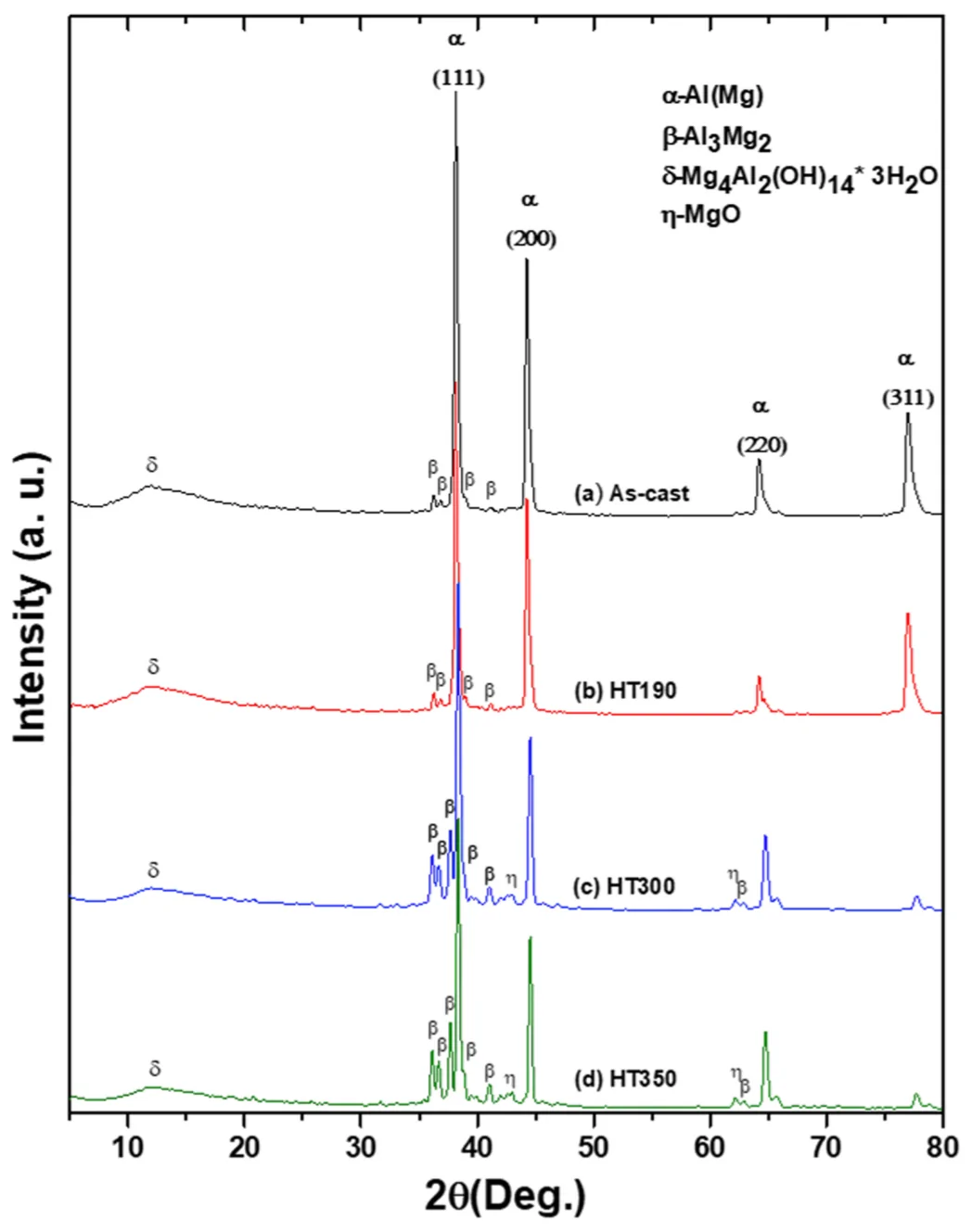
XRD patterns of the Al−20 wt.% Mg alloy as received and under corroded conditions at different metallurgical conditions of SS: (**a**) As−cast, (**b**) HT190 (**c**) HT300, and (**d**) HT 350.

Figure 12 presents the XRD patterns of the corrosion products formed on the alloy surfaces after electrochemical exposure to synthetic seawater under different metallurgical conditions (as−cast, HT190, HT300, and HT350). As shown in Figure 12a,b, the identified phases consist predominantly of α−Al(Mg), β−Al_3_Mg_2_, and the hydrated magnesium–aluminum hydroxide phase Mg_4_Al_2_(OH)_14_·3H_2_O [115,116]. The latter phase was identified according to the ICDD PDF No. 35-0964 and exhibits a rhombohedral crystal structure with space group R-3m (No. 166), with lattice parameters of *a* = 3.038 Å and *c* = 22.62 Å. The presence of Mg_4_Al_2_(OH)_14_·3H_2_O indicates the formation of hydrated hydroxide products associated with the oxidation and subsequent hydration of the alloy surface during electrochemical exposure.

The persistence of these phases across all conditions indicates that corrosion is strongly influenced by the interaction between the alloy microstructure and the corrosion products generated after exposure in synthetic seawater.

XRD analysis indicates the progressive oxidation and which forms microgalvanic couples with the surrounding α−Al(Mg) matrix (Figure 12). Therefore, localized dissolution occurs preferentially at α/β interfaces, grain boundaries, and regions adjacent to the intermetallic network, where microstructural heterogeneities promote anodic attack. This behavior is associated with both the intrinsic microstructural characteristics of the Al–20 wt.% Mg alloy and the modifications induced by the applied heat treatments [117]. The chemical reaction that can explain the formation of the corrosion products observed in Figure 12 is as follows:

Al_(*s*)_ + Al_3_Mg_2(*s*)_ + Al_2_MgO_4(*s*)_ + 9MgO_(*s*)_ + 30H_2_O_(*s*)_ + 4O_2(*g*)_ ⟶ 3Mg_4_Al_2_(OH)_14_·3H_2_O_(*s*)_ (6)

As reported by Meloni et al. [118], the precipitation behavior and growth kinetics of the β phase are highly dependent on alloy stoichiometry and thermal treatment parameters, particularly the annealing temperature [119]. Accordingly, the after-corrosion response of the alloy is governed by the degree of Mg supersaturation in the α matrix, the distribution of the β–Al_3_Mg_2_ phase, and the electrochemical interactions established between these constituents. The heat-treated samples, particularly HT300 and HT350 (Figure 12c,d), exhibit the η−MgO phase together with a more developed β phase, leading to increased microstructural heterogeneity. This heterogeneous distribution of phases promotes galvanic coupling, thereby accelerating localized corrosion.

Furthermore, the HT350 condition exhibited a more pronounced transformation of the aluminum-rich phases after exposure to synthetic seawater. This behavior suggests that the elevated thermal energy supplied during heat treatment promoted Mg redistribution, β−phase precipitation, and the formation of oxide phases, thereby reducing the stability of the protective surface film. Consequently, the passive Al_2_O_3_ layer becomes more susceptible to breakdown in chloride-containing environments, accelerating anodic dissolution and enhancing hydrolysis reactions [120]. The formation of MgO and hydrated magnesium–aluminum hydroxide phases further indicates active corrosion processes and continuous interaction between the alloy surface and the electrolyte.

The potentiodynamic polarization and EIS demonstrated a significant increase in corrosion susceptibility and elucidated the governing microgalvanic mechanisms, which may be indicative of enhanced hydrogen evolution as the dominant cathodic reaction. However, direct volumetric or generation-rate measurements were not performed in this study. The structural evolution of the β−Al_3_Mg_2_ phase suggests potentially favorable thermodynamic and kinetic conditions for the aluminum–water reaction, although this remains to be experimentally verified.

## 4. Conclusions

A heat-treated Al–20 wt.% Mg alloy was synthesized and characterized using OM, SEM–EDS, and XRD. Its electrochemical behavior was subsequently evaluated in synthetic seawater. Heat treatment significantly modified the electrochemical and corrosion behavior of the alloy by altering the precipitation, distribution, and morphology of the β−Al_3_Mg_2_ intermetallic phase. Diffusion-controlled magnesium redistribution changed the morphology and connectivity of the β−Al_3_Mg_2_ network, producing distinct microstructural states that governed corrosion susceptibility.

The HT190 condition exhibited the highest resistance to localized corrosion owing to a moderate redistribution of the β−Al_3_Mg_2_ phase, which reduced the anodic dissolution rate of β−Al_3_Mg_2_ and, consequently, the galvanically driven degradation of the surrounding α−Al(Mg) matrix. In contrast, the HT300 and HT350 conditions increased susceptibility to localized corrosion, promoting the formation of corrosion cavities, pitting, and an interconnected degradation network associated with the growth of the β−Al_3_Mg_2_ phase. Electrochemical impedance spectroscopy confirmed that heat treatment modified both the corrosion-product layer and the charge-transfer process, while microgalvanic coupling and passive-film destabilization were identified as the dominant mechanisms controlling corrosion evolution.

SEM–EDS and XRD analyses confirmed the formation of a heterogeneous corrosion-product layer composed primarily of aluminum and magnesium oxides and hydroxides, together with deposits originating from the synthetic seawater. The increased precipitation of the β−Al_3_Mg_2_ phase and the reduced stability of the passive film under the HT300 and HT350 conditions promoted an activated corrosion mechanism, thereby increasing the alloy reactivity. Therefore, the sample HT350 exhibited the highest electrochemical activation, which could potentially facilitate the aluminum–water reaction. 

In this research, it was possible to reduce the passivation of an Al−20 wt.% Mg alloy via heat treatment in aqueous medium (synthetic seawater), without the addition of acids or bases.

## Figures and Tables

**Table 1:** Denomination of samples. Sensitization (heat treatment).

Conditions	Sample			
	As-Cast	HT190	HT300	HT350
Heat treatment for 6 h (°C)	without HT	190	300	350

**Table 2:** Chemical composition (g/L) of synthetic seawater solution.

NaCl	MgCl_2_	Na_2_SO_4_	CaCl_2_	KCl	NaHCO_3_	KBr	H_3_BO_3_	SrCl_2_	NaF
24.53	5.2	4.09	1.16	0.695	0.201	0.101	0.027	0.025	0.003

**Table 3:** Descriptive statistics of the β−Al_3_Mg_2_ area fraction, n = 3.

Descriptive Statistics	Area Fraction (%)			
	As-Cast	HT190	HT300	HT350
β−Al_3_Mg_2_	12.461	33.285	41.658	36.100
	14.461	29.363	49.999	31.663
	16.107	38.374	50.576	38.874
$\bar{x}=\frac{\sum x_{i}}{n}\pm \frac{\sigma }{\sqrt{n}}$	14.343 ± 1.054	33.674 ± 2.608	47.411 ± 2.881	35.546 ± 2.100

**Table 4:** Electrochemical kinetic parameters calculated via Tafel extrapolation [108] of the Al–20 wt.% Mg alloy in synthetic seawater across as-cast and heat–treated conditions.

Sample	*OCP*	*E*_corr_	*i*_corr_	*β_a_*	*β_c_*	*E_pit*	*R_p_*
	mV	mV *_vs_*_SCE_	μA/cm^2^	mV/_decade_	mV/_decade_	mV	Ω·cm^2^
Without HT	−960.4	−1222	2.04	46.91	59.58	−831.3	400.000
HT190	−947.8	−1148	2.37	53.86	70.70	−839.8	1111.11
HT300	−915.1	−1232	2.13	47.09	60.21	−851.7	434.780
HT350	−1037.7	−1154	2.58	18.02	20.56	−836.9	285.710

**Table 5:** Electrochemical impedance spectroscopy component values for the Al−20 wt.% Mg alloy at various heat-treatment conditions.

Parameters		As−Cast	HT190	HT300	HT350
**Rs (Ω∙cm^2^)**		11.2	12.83	12.25	18.98
**Q_r1_ (Ω^−1^∙s^n^∙cm^−2^)**	Y_r1_	6.04 × 10^−4^	1.34 × 10^−4^	5.28 × 10^−5^	4.99 × 10^−5^
	n_r1_	0.54	0.72	0.80	0.79
**R_rl_ (Ω∙cm^2^)**		28.65	4283	2853	629.3
**Q_dl_ (Ω^−1^∙s^n^∙cm^−2^)**	Y_r2_	1.09 × 10^−4^	1.51 × 10^−3^	7.60 × 10^−5^	5.40 × 10^−5^
	n_r2_	0.82	0.87	1	0.91
**R_ct_ (Ω∙cm^2^)**		3537	4455	2985	1115
**W,Yo (S∙s^0.5^∙cm^2^)**	Ws	-	−	4.59 × 10^−4^	1.62 × 10^−3^
**Chi−squared (adimentional)**		2.4 × 10^−3^	1.6 × 10^−3^	2.4 × 10^−3^	3.6 × 10^−3^

## Data Availability

The original contributions presented in this study are included in the article. Further inquiries can be directed to the corresponding authors.

## Abbreviations

The following abbreviations are used in this manuscript:

EIS	Electrochemical Impedance Spectroscopy
EDS	Energy-Dispersive X-ray Spectroscopy
EEC	Equivalent Electrical Circuit
HT	Heat Treatment
HE	Hydrogen Embrittlement
IGC	Intergranular Corrosion
OCP	Open-Circuit Potential
OM	Optical Microscopy
Epit	Pitting Potentials
SCE	Saturated Calomel Electrode
SEM	Scanning Electron Microscope
SCC	Stress Corrosion Cracking
SS	Synthetic Seawater
XRD	X-ray Diffraction
